# Effectiveness of Immediate Pharmacological Versus Electrical Cardioversion on Hemodynamic Stability and Rhythm Restoration in Patients With Refractory Atrial Fibrillation: A Systematic Review

**DOI:** 10.7759/cureus.86747

**Published:** 2025-06-25

**Authors:** Paulina Elizabeth Cisneros Clavijo, Mishell Estefania Zambrano Mila, Cesar Antonio Cervantes Ruiz, David Sebastian Ramirez Calvillo, Leonardo Alvarado Rangel, Daniel Alexander Robles Gutiérrez, Carlos Antonio Ramirez Arbelaez, Hannia Sarali Mares Cárdenas, Sophia Alejandra Aguirre Castro

**Affiliations:** 1 Endovascular Surgery, Enrique Garcés Hospital, Quito, ECU; 2 Hemodynamics and General and Interventional Cardioangiology, Pontifical Catholic University of Ecuador, Quito, ECU; 3 Medical Department, Ministry of Public Health, Quito, ECU; 4 Internal Medicine, Mexican Social Security Institute, San Luis Potosí, MEX; 5 Medical Department, Roosevelt Basic Hospital, Latacunga, ECU; 6 Medical Department, Ministry of Public Health, Barranquilla, COL; 7 Mexican Social Security Institute, Internal Medicine Resident, Mexico, MEX; 8 Medical Department, Ministry of Public Health, Loja, ECU

**Keywords:** atrial fibrillation, electrical cardioversion, emergency service, hemodynamic stability, pharmacologic cardioversion

## Abstract

Atrial fibrillation (AF) is the most frequently encountered arrhythmia in emergency departments (EDs), often requiring urgent rhythm control when rate control fails. This systematic review and meta-analysis compare electrical cardioversion (ECV) and pharmacological cardioversion (PCV) in adult patients with both new-onset AF (NOAF) and refractoryAF, defined in the included studies as AF unresponsive to initial rate control and necessitating immediate rhythm intervention due to ongoing symptoms or hemodynamic instability. A comprehensive literature search was conducted in PubMed, Google Scholar and Cochrane Library. Eligible studies included randomized or observational trials comparing ECV and PCV in emergency settings. Screening and selection were performed independently and in duplicate. Eight studies (n=1,561) were included. ECV generally showed higher rhythm restoration rates, especially in persistent AF (e.g., 59.1% vs 12.5%; p=0.002). However, the pooled odds ratio (OR) was 1.31 (95% confidence interval (CI): 0.55-3.13; p=0.55), indicating no significant difference. Wide CIs and high heterogeneity (I²=88%) reflect imprecision and possible underpowering. Findings in persistent AF were not from a predefined subgroup analysis and should be interpreted cautiously. Three studies assessed discharge rates; pooled analysis showed no significant difference (OR=0.66, 95% CI: 0.24-1.79; p=0.42; I²=77%), despite individual studies favoring ECV for earlier discharge. Both strategies were safe, with no deaths or major complications. Safety assessments included hypotension, bradyarrhythmias, and procedural complications. Minor adverse events were rare and transient. In conclusion, ECV and PCV are both effective and safe for managing refractory AF in emergency settings. Clinical choice should consider patient-specific factors and provider experience.

## Introduction and background

Atrial fibrillation (AF) is the most prevalent form of cardiac arrhythmia, characterized by disorganized electrical activity in the atria that leads to ineffective atrial contractions and an irregularly rapid ventricular response [1]. It is classified as a supraventricular tachyarrhythmia and may present as either new-onset AF (NOAF), defined as an episode lasting less than seven days, or as persistent or chronic AF lasting more than seven days [1,2]. One of the major clinical concerns associated with AF is its predisposition to thrombus formation due to turbulent blood flow in the atria, increasing the risk of embolic stroke. AF is the most common cardiac cause of stroke [2].

Common risk factors for AF include increasing age, hypertension, structural heart disease, congenital heart defects, chronic pulmonary disease, alcohol consumption, and other comorbidities. Clinical manifestations range from asymptomatic to severe, including palpitations, chest discomfort, dyspnea, dizziness, fatigue, and diaphoresis [3].

The incidence of AF increases markedly with age, with a global prevalence estimated at 1%, rising to approximately 9% in individuals over the age of 75. By 2050, the global burden of AF is expected to double or even triple [1]. Lifetime risk approaches 22% by age 80. AF is more prevalent in men and in white populations compared to Black individuals [4,5].

This review focuses on the acute management of both NOAF and refractory AF in the emergency setting, where immediate decisions must be made regarding rhythm versus rate control. Rhythm control aims to restore normal sinus rhythm and is often preferred in younger patients or those with severe symptoms. Rate control, on the other hand, prioritizes heart rate regulation to alleviate symptoms and improve quality of life without restoring sinus rhythm [6].

Among rhythm control strategies, electrical cardioversion (ECV) is generally the first-line treatment for hemodynamically unstable patients. For stable patients with NOAF or refractory AF, however, practice varies between using ECV directly ("shock-only" approach) or attempting pharmacological cardioversion (PCV) first, possibly followed by ECV if initial drug therapy fails (referred to as the "drug-shock" strategy) [6,7]. ECV has a higher success rate-typically 80-89%, particularly in NOAF and post-PCV failure cases, though it requires sedation and carries procedural risks [8]. In contrast, PCV is non-invasive and avoids anesthesia but has a lower success rate (around 50%) and carries a risk of antiarrhythmic side effects.

Two previous meta-analyses compared ECV and PCV in the treatment of NOAF, including mixed protocols where ECV followed failed PCV [9,10]. While both strategies showed comparable sinus rhythm restoration rates, ECV was associated with a lower incidence of hypotension. However, these earlier reviews were limited by small sample sizes (only two-four studies), methodological heterogeneity, inconsistent outcome definitions, and limited reporting of safety and secondary outcomes. To address these gaps, this systematic review and meta-analysis aims to provide an updated, comprehensive compare son of ECV versus PCV in the emergency management of both NOAF and refractory AF, focusing on efficacy (rhythm restoration), hemodynamic stability, and safety outcomes.

## Review

Methodology

Study Design

This systematic review and meta-analysis was conducted in accordance with the Preferred Reporting Items for Systematic Reviews and Meta-Analyses (PRISMA) 2020 guidelines [11]. The review aimed to synthesize current evidence comparing ECV and PCV for the treatment of both NOAF and refractory AF in emergency settings.

Search Strategy and Data Sources

A comprehensive and systematic search was carried out using five major databases: PubMed, Cochrane Library, and Google Scholar. While PubMed and Cochrane were used for structured searches of peer-reviewed articles, Google Scholar was used specifically to identify relevant grey literature and ensure no potentially eligible studies were overlooked. The search combined MeSH terms and free-text keywords with Boolean operators, using terms such as “atrial fibrillation” OR “AF” AND “electrical cardioversion” OR “pharmacological cardioversion” AND “emergency department” OR “ED.” Filters were applied to limit results to English-language studies involving human adults (≥18 years) with full-text availability. The literature search covered studies published between January 1, 2000, and February 29, 2024, with the final search conducted on March 1, 2024.

Inclusion and Exclusion Criteria

Eligible studies included randomized controlled trials (RCTs) and observational comparative studies that examined the effectiveness and safety of PCV versus ECV in adult patients presenting to emergency departments (EDs) with either NOAF or refractory AF. Refractory AF was operationally defined as AF not responsive to initial rate or rhythm control strategies, including failure of prior cardioversion attempts, as described in the respective study protocols. Only studies that reported at least one clinical outcome, such as rhythm restoration, discharge status, and hemodynamic stability, were considered. Studies were excluded if they lacked a comparative group, were not in English, or were designed as case reports, conference abstracts, editorials, letters, or reviews. Additionally, studies were excluded if their methodology lacked sufficient detail or if outcome data were unclear or incomplete. While no strict sample size threshold was used for exclusion, studies with very small sample sizes (<10 participants per group) were flagged for sensitivity analysis.

Selection of Studies

Full texts of eligible studies were reviewed for inclusion. References of included studies were also screened to identify additional relevant articles. In cases of duplicate data, the most recent and comprehensive study was selected.

Data Extraction and Management

Extracted data included study characteristics (authors, year, design), patient demographics, intervention and comparator details, primary outcomes (rhythm restoration), secondary outcomes (discharge rates, hemodynamic stability), and effect sizes (odds ratios (ORs), 95% confidence intervals (CIs)). Any disagreements were resolved by consensus. Data were compiled in Covidence and analyzed using Review Manager (RevMan) version 5.4.

Assessment of Risk of Bias in Included Studies

To assess the methodological quality of the included studies, RCTs were evaluated using the Cochrane Risk of Bias (RoB) 2.0 tool, while observational studies were assessed using the Newcastle-Ottawa Scale (NOS). These assessments were independently conducted by two reviewers. In the event of disagreement, discrepancies were discussed and resolved by mutual agreement or consultation with a third reviewer. While all included studies met minimal quality thresholds, those rated at high risk of bias were noted and accounted for during sensitivity analyses to evaluate the impact on overall findings.

Assessment of Heterogeneity

Heterogeneity across included studies was evaluated statistically using the I² statistic, where values greater than 50% were considered indicative of substantial heterogeneity. The chi-squared (χ²) test was also used, with a p-value less than 0.10 regarded as statistically significant. In addition to statistical methods, clinical and methodological heterogeneity were also assessed qualitatively, based on differences in patient populations, intervention protocols, and study designs.

Assessment of Reporting Biases

To evaluate potential publication bias, funnel plots were generated for outcome analyses that included ten or more studies. Due to the limited number of studies available for some comparisons, the interpretation of funnel plot asymmetry was approached with caution. Reporting biases were also considered during data interpretation, especially when outcomes were inconsistently reported across studies.

Data Synthesis

Data synthesis was carried out using RevMan version 5.4. Pooled ORs with 95% CIs were calculated for dichotomous outcomes. A fixed-effect model was used for meta-analyses with low heterogeneity (I²<50%) and low clinical variability. When substantial heterogeneity or variability in study designs was present, a random-effects model was applied to better account for differences between studies.

Results

**Figure 1:**
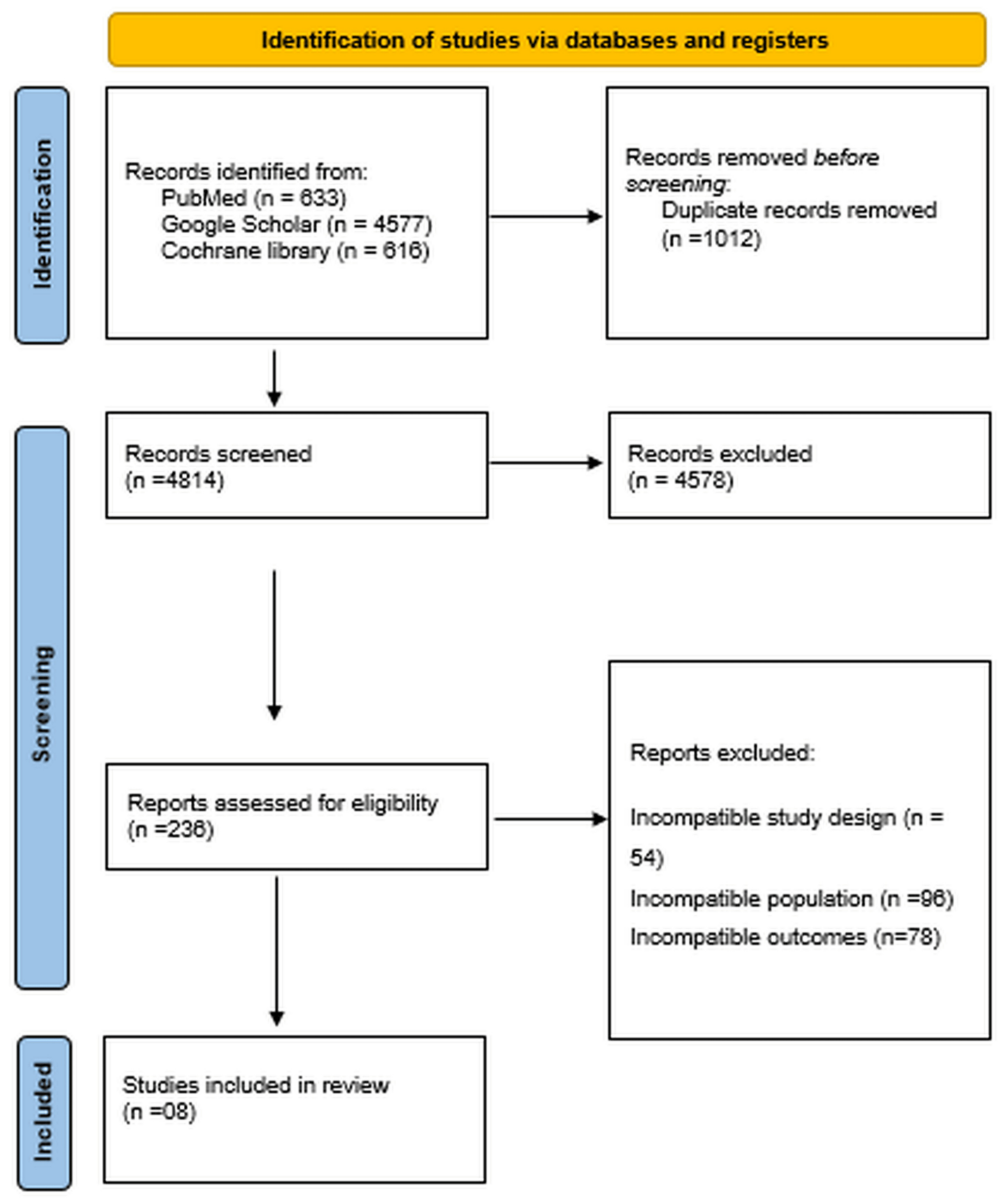
PRISMA flow diagram PRISMA: Preferred Reporting Items for Systematic Reviews and Meta-Analyses

**Figure 2:**
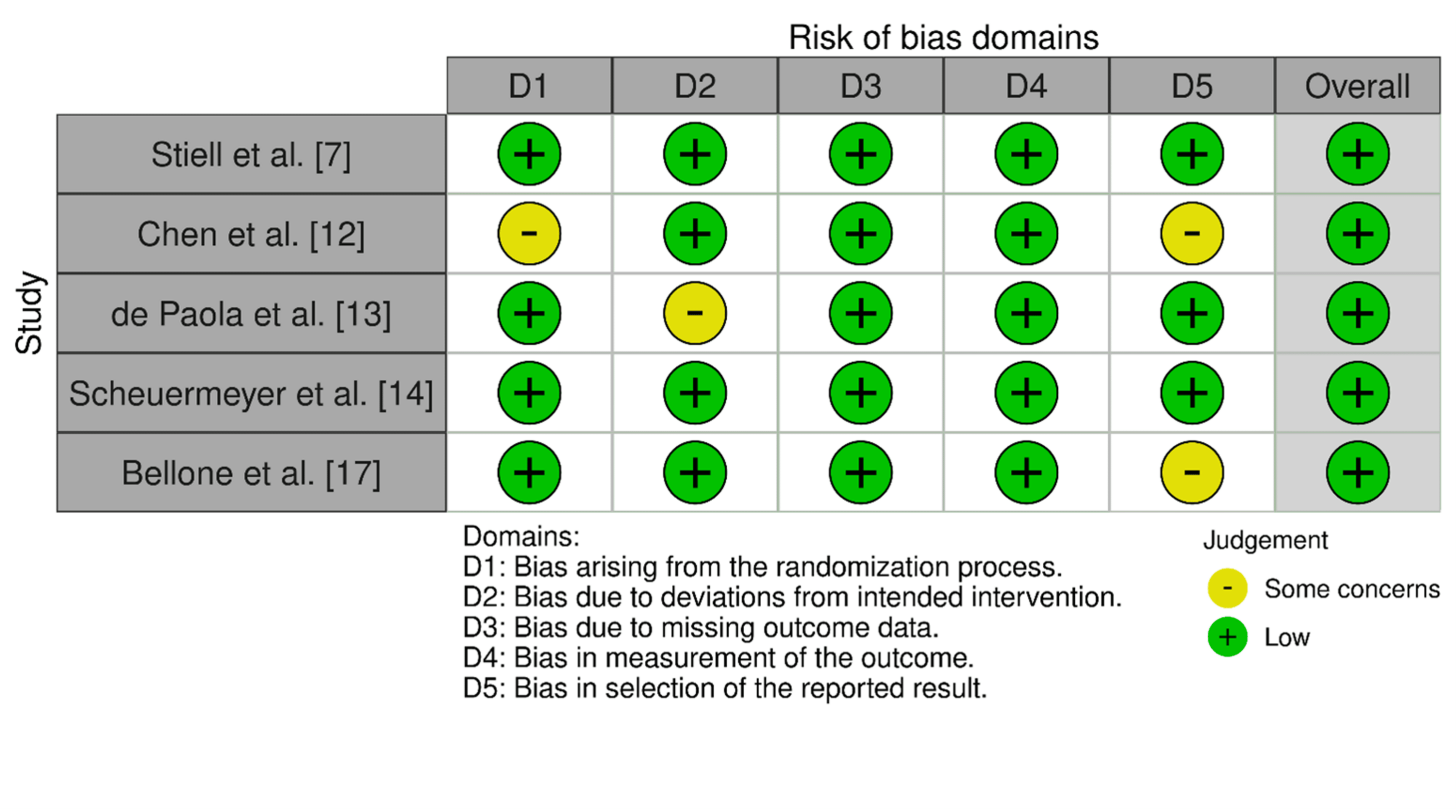
Quality assessment of RCTs by RoB 2.0 RCT: Randomized controlled trials; RoB: Risk of Bias

Database searches in PubMed, Google Scholar and the Cochrane Library yielded 5826 entries. 4,814 studies were left for title and abstract screening after duplicates were eliminated. The eligibility of 236 full-text publications was then evaluated using predetermined inclusion and exclusion criteria. The final qualitative and quantitative synthesis included eight studies that satisfied all inclusion criteria. The PRISMA flow diagram (Figure 1) shows the whole research selection procedure. Characteristics and outcomes are stated in Table 1. Quality assessment of cohort studies is done by the NOS (Table 2), and RCT by RoB 2.0 (Figure 2).

**Table 1:** Characteristics and outcomes of the studies included RCT: Randomized controlled trial; ED: Emergency department; AF: Atrial fibrillation; ECV: Electrical cardioversion; PCV: Pharmacological cardioversion; DCC: Direct-current cardioversion; CHADS2: Congestive heart failure, hypertension, age ≥75 years, diabetes mellitus, and prior stroke or transient ischemic attack (2 points); NS: Not significant

Author (Ref)	Year	Study Design	Population	Male/Female	Intervention	Control	Results with Stats	Adverse Events	Conclusion
Stiell et al. [7]	2020 (online 2013)	Partial factorial RCT	396 ED patients with acute AF	Drug-shock: 134/70 Shock: 126/66	Procainamide IV (15 mg/kg/30 min)+ECV if needed	ECV alone	Conversion: 96% (drug-shock) vs 92% (shock-only); p=0.07.52% in drug-shock converted without shocks. Discharge: 97% vs 95%; p=0.60. Pad position outcome: 94% vs 92%; p=0.68.	No serious adverse events. 52% avoided sedation in procainamide group.	Both methods safe and effective.Drug-shock avoided sedation in 50% of cases.No difference due to pad position.
Dankner et al. [8]	2009	Retrospective cohort	374 stable AF patients in ED	Chemical: 58/26 Electrical: 60/26	Procainamide infusion+ECV if needed	ECV followed by procainamide if needed	Success rates of rhythm restoration: DCC 78.2% vs pharmacological 59.2% vs conservative 37.9%; p<0.001. Discharge: DCC 52.9% vs pharmacological 47.9% vs conservative 32.1%; p<0.01. Adjusted OR: DCC 6.00 vs pharmacological 2.47.	3.4% rehospitalization within 14 days (treatment-related).	DCC most effective with low complication rate. Supports DCC use in stable AF in ED.
Chen et al. [12]	2013	Prospective RCT	115 post-valve replacement patients with permanent AF	Not specified	Amiodarone, Captopril, Simvastatin (3-month oral therapy)	Direct-current synchronized ECV	Conversion: ECV 98.3% vs pharmacological 26.8%. Recurrence: electrical 3.4% vs pharmacological 6.7%. ECV statistically superior.	No deaths or severe complications in either group. 3-month follow-up.	ECV significantly superior for restoring sinus rhythm in post-valve AF with normalized heart size.
de Paola et al. [13]	2003	Multicenter RCT	139 patients with persistent AF (<6 months)	74/65	Initial antiarrhythmic therapy (various drugs)	ECV	Initial success: 74% (chemical) vs 73% (ECV); p=0.95. Overall success: 96% (chemical) vs 84% (ECV); p=0.0016. Cost: $1,240 (chemical) vs $1,917 (ECV); p=0.002.	5% life-threatening complications (all in chemical group with structural heart disease).	Chemical cardioversion more cost-effective and slightly more successful. Use caution in structural heart disease.
Scheuermeyer et al. [14]	2019	Multicenter RCT	84 ED patients with AF<48h, CHADS2=0–1	57/32	Antazoline+Propafenone (PCV)	ECV	Sinus rhythm: 99% overall. Discharge<4h: 67% (ECV-first) vs 32%; p=0.001. Median ED stay: 3.5 h (ECV) vs 5.1 h; p<0.001.	No strokes, deaths, or significant AEs. 30-day follow-up.	Both effective and safe. ECV-first reduced ED stay and improved early discharge.
Klocek et al. [15]	2024	Retrospective cohort	89 patients with AF during cryoablation	169/205	Pharmacological therapy (various drugs)	DCC	Overall success: ECV 72.1% vs drug 43.5%; p=0.01. Persistent AF: ECV 59.1% vs drug 12.5%; p=0.002. Paroxysmal AF: 85.6% vs 77.3%; p=0.7.	Not reported	ECV significantly superior, especially in persistent AF. No major difference in paroxysmal AF.
Valencia Martín et al. [16]	2002	Comparative cohort study	230 AF patients (>48h duration)	112/118	ECV	Quinidine (PCV)	Success: Electrical 77%, pharmacological 81% (NS). Second ECV attempt after failed PCV: 61%. AF duration<8 weeks predicted better outcomes; p<0.01.	No embolic events. 2 minor electrical disturbances.	Both methods effective. Quinidine as effective as ECV but with longer hospital stay.
Bellone et al. [17]	2012	Prospective RCT	247 acute AF patients (<48h), stable	Not specified	Not specified	Not specified	Success: ECV 89.3% vs PCV 73.8%; p=0.02. ED stay: ECV 180 min vs PCV 420 min; p<0.001.	Minor and transient AEs in both groups.	ECV more effective and reduced ED stay.Both strategies were safe.

**Table 2:** Quality assessment of the reviewed studies by NOS NOS: Newcastle-Ottawa Scale

Study	Representativeness of the exposed cohort	Selection of the non-exposed cohort	Ascertainment of exposure	Demonstration that outcome of interest was not present at start of study	Compare ability of cohorts on the basis of the design or analysis	Assessment of outcome (1)	Was follow-up long enough for outcomes to occur	Adequacy of follow up of cohorts	Representativeness of the exposed cohort
Dankner et al. [8]	1	1	1	0	2	1	1	1	1
Klocek et al. [15]	1	1	1	1	2	1	1	1	1
Valencia Martín et al. [16]	1	1	1	0	2	1	1	1	1

Rhythm Restoration

ECV generally demonstrated higher success rates compared to pharmacological methods, particularly in studies involving persistent AF. Trials by Dankner et al., Bellone et al., and Chen et al. reported significantly greater rates of immediate sinus rhythm restoration with direct-current cardioversion (DCC) than with PCV. Reported success rates were 78.2% vs 59.2% (p<0.001), 89.3% vs 73.8% (p=0.02), and 98.3% vs 26.8% (p<0.001), respectively, favoring the electrical group [8,17,12]. In patients with persistent AF, Klocek et al. also observed superior outcomes with DCC (59.1% vs 12.5%; p=0.002), whereas outcomes were more comparable in paroxysmal AF [15].

However, when applied within structured or step-wise protocols, both strategies showed high and often comparable success. In the Rate Control versus Electrical Cardioversion for Persistent Atrial Fibrillation II (RAFF2) trial, 96% of patients undergoing a sequential pharmacological-electrical protocol (procainamide followed by DCC if needed) achieved conversion, compared to 92% in the electrical-only arm (p=0.07). Notably, more than half converted with procainamide alone, thus avoiding shocks [7]. Similarly, de Paola et al. found equivalent initial conversion rates with pharmacological and electrical methods (74% vs 73%; p=0.95), although the overall success of the pharmacological-first sequential strategy was higher (96% vs 84%; p=0.0016) [13]. Valencia Martín et al. reported near-equivalent success between pharmacological (81%) and electrical (77%) interventions, and Scheuermeyer et al. documented near-universal success (99%) regardless of strategy used [14,16].

A meta-analysis of eight studies (n=1,561) comparing PCV versus ECV in ED patients with recent-onset or symptomatic AF showed no statistically significant difference in achieving sinus rhythm. The pooled OR was 1.31 (95% CI: 0.55 to 3.13; Z=0.60; p=0.55), with substantial heterogeneity (I²=88%, p<0.00001). Variation in drug regimens (e.g., procainamide, flecainide, amiodarone), patient characteristics (e.g., duration of AF, comorbidities), and protocol structure likely contributed to the heterogeneity. Additionally, differing definitions of “conversion success” (e.g., rhythm restoration during ED stay vs within one-two hours) and “discharge” criteria may have influenced comparability.

Some studies appeared to favor one modality over the other. For example, Bellone et al. reported an OR of 0.34 (95% CI: 0.17-0.68), indicating greater odds of success in the electrical group when the pharmacological group is the reference. In contrast, Scheuermeyer et al. found an OR of 11.86 (95% CI: 0.63-221.63), favoring pharmacological strategy in their cohort. Interpretation of ORs must consider the reference group; here, values above 1 favor PCV, and values below 1 favor electrical. Inconsistencies in reporting reference groups across studies may risk misinterpretation and bias.

**Figure 3:**
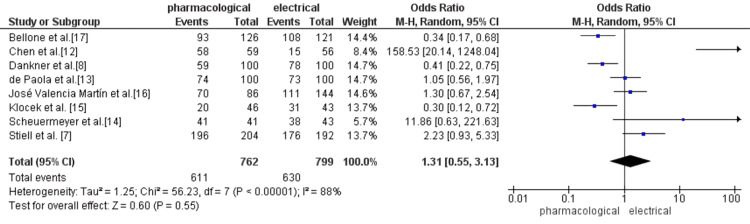
Forest plot of rhythm restoration

Given the inclusion of fewer than 10 studies, assessment of publication bias using funnel plots or statistical tests remains limited in validity. Moreover, small-study effects and methodological differences may contribute to overestimation or underestimation of true treatment effects (Figure 3).

Hemodynamic Stability and Adverse Events

Both strategies were safe with low adverse event rates. No serious complications, strokes, or deaths were reported in either treatment group in the studies by Stiell et al., Scheuermeyer et al., Bellone et al., and Chen et al., indicating a favorable safety profile for both strategies in acute settings [7,12,14,17]. Minor adverse events were infrequent and transient. For instance, Valencia Martín et al. reported only two cases of minor electrical disturbances [16]. Dankner et al. reported a 3.4% 14-day readmission rate, and de Paola et al. observed a 5% complication rate in patients with structural heart disease treated pharmacologically [8,13]. Notably, Stiell et al. found that 52% of patients avoided sedation by converting with medication alone [7].

ED Length of Stay and Discharge Rate

Bellone et al. reported a significantly shorter ED stay in the electrical group (180 minutes) compared to the pharmacological group (420 minutes; p<0.001) [17]. Similarly, Scheuermeyer et al. demonstrated that 67% of patients in the electrical-first group were discharged within four hours, versus only 32% in the chemical-first group (p=0.001), with a median length of stay (LOS) of 3.5 hours vs 5.1 hours (p<0.001) [14]. Dankner et al. also noted higher discharge rates with electrical (53%) than pharmacological (48%) or conservative management (32%) [8]. The RAFF2 trial showed high discharge rates in both treatment arms, with no statistically significant difference (97% for drug-shock and 95% for electrical-only; p=0.60), suggesting that both strategies can be safely and effectively applied to facilitate early discharge [7].

**Figure 4:**
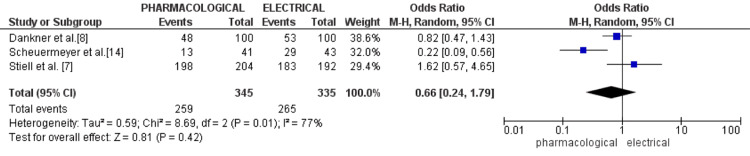
Forest plot of discharge rates

The forest plot illustrates a meta-analysis comparing the discharge rates following immediate PCV versus ECV in patients with refractory AF in the ED. Data from three studies, encompassing a total of 680 patients (345 in the pharmacological group and 335 in the electrical group), were analyzed. The pooled OR was 0.66 with a 95% CI of 0.24 to 1.79, suggesting no statistically significant difference in discharge rates between the two treatment modalities (Z=0.81, p=0.42). Heterogeneity among the studies was substantial (I²=77%, p=0.01), indicating variability in study outcomes. While individual studies reported mixed results, Scheuermeyer et al. showed a significant benefit for ECV (OR 0.22, 95% CI (0.09, 0.56)), other studies did not demonstrate statistically significant differences (Figure 4) [14].

Publication Bias

**Figure 5:**
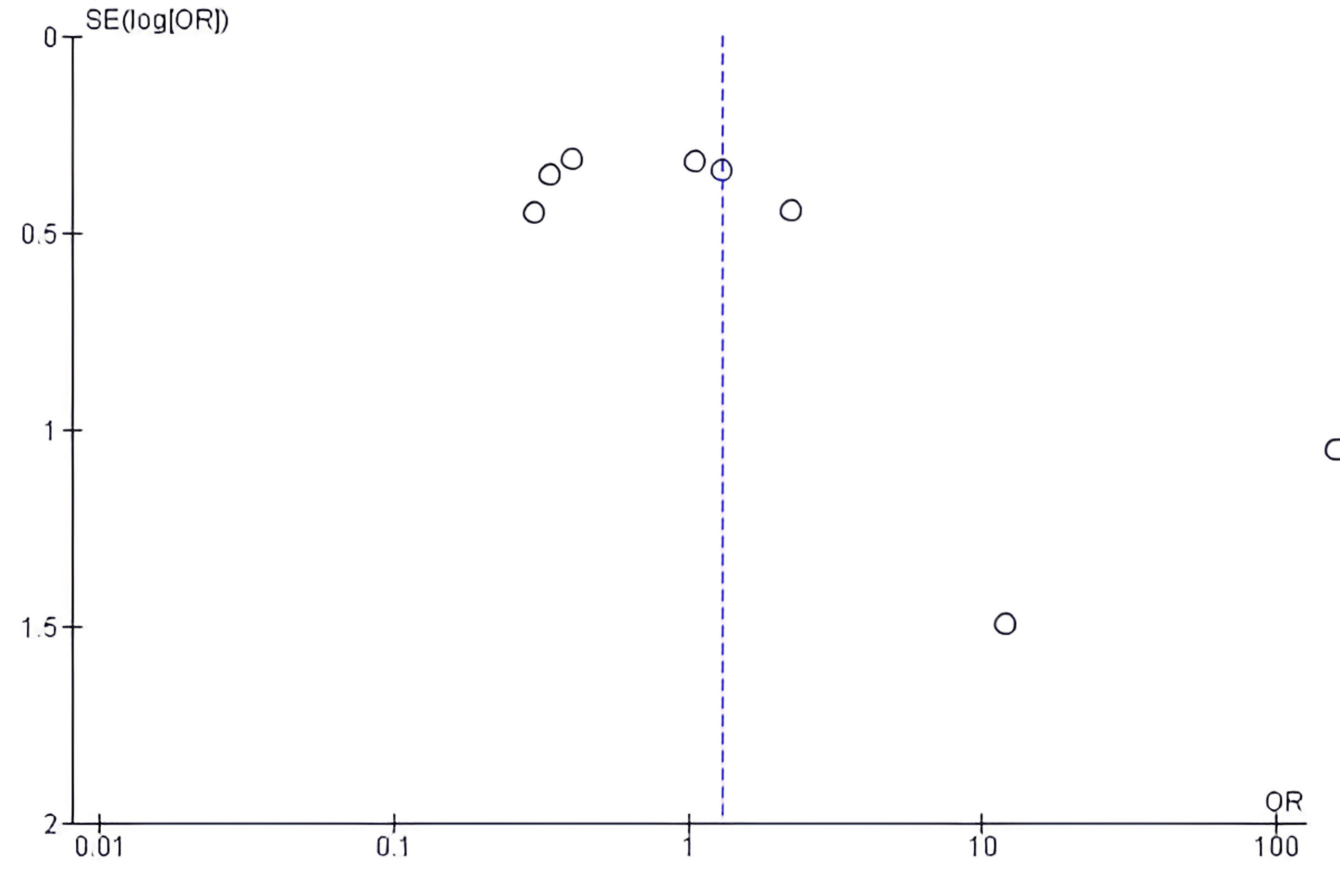
Funnel plot showing publication bias

The funnel plot presented evaluates potential publication bias in the meta-analysis comparing the effectiveness of immediate PCV versus ECV. The plot displays a relatively asymmetrical distribution of studies, with some studies lying outside the expected funnel-shaped region, particularly on the right side, with higher standard errors. This visual asymmetry suggests a possible presence of publication bias or small-study effects, where smaller studies with larger effect sizes may be over-represented. However, interpretation should be made cautiously due to the limited number of included studies (fewer than 10), which reduces the reliability of funnel plot assessments (Figure 5).

Discussion

The comparative efficacy of PCV versus ECV in managing acute AF remains a nuanced issue, influenced by AF duration, patient comorbidities, and care protocols. While numerous studies report higher immediate sinus rhythm restoration with ECV, particularly in persistent AF, interpretation must consider the quality and design of individual studies. High-quality RCTs, such as RAFF2, provide stronger evidence than smaller observational cohorts like those by Bellone et al., Chen et al., or Dankner et al., which, although supportive of ECV, carry greater risk of bias [7,8,12,17] .

In persistent AF, where spontaneous conversion is less likely and pharmacological responsiveness is diminished, ECV often demonstrates superior outcomes. This was evident in Klocek et al. (ECV: 59.1% vs PCV: 12.5%; p=0.002) and Chen et al., where ECV restored sinus rhythm in 98.3% of patients vs only 26.8% in the PCV group [12,15]. These findings were reinforced by Bellone et al. (89.3% vs 73.8%; p=0.02) and Dankner et al. (78.2% vs 59.2%; p<0.001) [8,17] . While these absolute differences, ranging from 16% to over 70%, appear clinically meaningful, their impact on outcomes such as hospital stay, recurrence, and quality of life warrants deeper exploration.

Conversely, RCTs like RAFF2, which employed a drug-shock sequential strategy (procainamide followed by ECV if needed), found no statistically significant difference in rhythm restoration rates compared to ECV alone (96% vs 92%; p=0.07), but notably over half of patients (52%) converted with pharmacological therapy alone, thereby avoiding electrical shocks and sedation [7]. This strategy offers a practical compromise: rapid pharmacological conversion where effective, with ECV as backup for non-responders-potentially optimizing patient safety, comfort, and ED efficiency. Similarly, de Paola et al. reported that allowing non-responders in the PCV arm to undergo ECV led to an overall higher cumulative conversion success (96% vs 84%; p=0.0016) [13].

From a patient-centered perspective, the avoidance of sedation or general anesthesia, particularly relevant in elderly or comorbid populations, is a major advantage of pharmacological or step-wise approaches. ECV is generally safe, but procedural sedation carries risks of hypotension, respiratory depression, and aspiration. These concerns were underscored by Stiell et al., who found that over half the patients avoided sedation altogether with the drug-shock strategy [7]. While no study reported major complications like stroke or death, de Paola et al. noted a slightly higher complication rate in PCV-treated patients with structural heart disease, albeit without statistical significance [13]. Safety overall was favorable across studies, with only minor, self-limiting adverse effects reported [7,13,14,17].

The meta-analysis included eight studies totaling over 1,500 patients and found no statistically significant difference in rhythm conversion rates between PCV and ECV (pooled OR=1.31 (95% CI: 0.55-3.13), p=0.55). This lack of significance may reflect true clinical equivalence or may be masked by substantial heterogeneity (I²=88%). Sources of this heterogeneity include differences in patient age, AF chronicity, time to intervention, drug type (e.g., amiodarone, procainamide), sedation use, and institution-level factors such as ED cardioversion protocols and provider experience. For example, Scheuermeyer et al. and Bellone et al. conducted studies in high-throughput EDs with streamlined ECV protocols, potentially explaining their shorter ED stays and higher discharge rates for ECV-treated patients [14,17].

Institutional workflows can profoundly affect outcomes like LOS and discharge rate-critical metrics in EDs where efficiency is essential. While Dankner et al., Bellone et al., and Scheuermeyer et al. found ECV associated with shorter ED stays, RAFF2 showed no significant difference between the groups [7,8,14,17]. These findings suggest that organizational infrastructure, not only treatment modality, shapes patient flow, and may explain some of the observed inter-study variability (reflected in high I² values).

Despite the focus on rhythm conversion and discharge, clinical relevance must extend beyond immediate procedural success. None of the included studies systematically reported AF recurrence, symptom relief, or patient-reported outcomes (PROs), such as quality of life or treatment satisfaction, which are critical dimensions of care that affect long-term management. Future trials should integrate these endpoints to provide a more holistic understanding of treatment impact.

There is also the potential for publication bias, particularly given the small number of included studies and visual asymmetry in the funnel plot. This bias could inflate the perceived efficacy of ECV if studies with neutral or negative findings remained unpublished. Alternatively, true equivalence between strategies may be obscured due to selective reporting or underpowering of smaller trials. The reliance on English-language studies may further compound this bias.

Limitations

This review has several limitations. First, it pooled data from both RCTs and observational studies, which may differ in rigor and susceptibility to confounding. Second, variation in protocols-including drug types, doses, ECV techniques, timing of interventions, and criteria for success-limited comparability and likely contributed to heterogeneity. Third, the definitions of AF varied: While some studies targeted acute AF (<48 hours), others included persistent or chronic AF, affecting generalizability. Additionally, adverse events and secondary outcomes such as hemodynamic stability and discharge criteria were inconsistently reported, precluding robust subgroup analyses.

To strengthen future evidence, high-quality multicenter RCTs are needed that address current gaps in the literature. These trials should stratify patients based on key clinical variables such as AF duration (acute vs persistent), age, and the presence of comorbidities to allow for more precise subgroup analyses. Direct comparisons between step-wise strategies (e.g., drug-shock) and single-modality approaches would provide greater clarity on the most effective and safest treatment paths. Importantly, future studies should go beyond immediate rhythm restoration and include long-term outcomes such as AF recurrence, symptom control, and patient-reported quality of life to capture the full clinical relevance of each intervention. Additionally, standardized reporting of secondary outcomes, such as ED LOS, discharge timing, adverse events, and the need for sedation, would improve comparability across studies. Such comprehensive trial designs would better inform evidence-based, individualized treatment strategies in both emergency and outpatient care.

## Conclusions

ECV and PCV are both effective strategies for rhythm restoration in acute AF management within emergency settings, with comparable overall success rates. ECV tends to yield higher immediate success, particularly in patients with persistent or long-standing persistent AF, as defined by European Society of Cardiology guidelines. PCV, especially when used in a sequential drug-shock strategy may reduce the need for procedural sedation and anesthesia, offering a clinical advantage, particularly in elderly or comorbid patients. While some studies suggest shorter ED stays with ECV, findings on discharge outcomes remain mixed and should be interpreted with caution due to the high heterogeneity observed in meta-analyses. These inconsistencies likely stem from differences in patient selection, treatment protocols, and institutional practices across studies. Therefore, the conclusion that a sequential approach is most practical and effective should be contextualized by available evidence and recognized as not universally applicable. An individualized, patient-centered treatment strategy, accounting for clinical profile, AF type, and institutional capacity, remains the most prudent recommendation, pending further high-quality comparative trials.
